# Deterioration risk of dryland earthen heritage sites facing future climatic uncertainty

**DOI:** 10.1038/s41598-020-73456-8

**Published:** 2020-10-02

**Authors:** Jenny Richards, Richard Bailey, Jerome Mayaud, Heather Viles, Qinglin Guo, Xudong Wang

**Affiliations:** 1 School of Geography and the Environment, University of Oxford, Oxford, OX1 3QF UK; 2 Dunhuang Academy, Dunhuang, 736200 China; 3 The Palace Museum, Beijing, 100009 China

**Keywords:** Environmental impact, Climate-change impacts, Climate and Earth system modelling

## Abstract

Uncertainties over future climatic conditions pose significant challenges when selecting appropriate conservation strategies for heritage sites. Choosing effective strategies is especially important for earthen heritage sites located in dryland regions, as many are experiencing rapid environmentally-driven deterioration. We use a newly developed cellular automaton model (ViSTA-HD), to evaluate the environmental deterioration risk, over a 100-year period, under a range of potential climate and conservation scenarios. Results show increased wind velocities could substantially increase the overall deterioration risk, implying the need for wind-reducing conservation strategies. In contrast, predicted increases in rainfall are not likely to increase the overall deterioration risk, despite greater risk of rain-driven deterioration features. Of the four conservation strategies tested in our model, deterioration risk under all climatic scenarios was best reduced by increasing the coverage of natural, randomly-distributed vegetation to 80%. We suggest this approach could be an appropriate long-term conservation strategy for other earthen sites in dryland regions.

## Introduction

Many earthen heritage sites (historic structures formed of earthen materials) are experiencing extensive deterioration that is driven by climatic factors such as wind and rain^1–7^. For these sites to be passed on to future generations, effective conservation strategies are urgently needed. Such strategies must:

(i) address the complexity of interactions between the environment and earthen heritage sites^4–6,8^;
(ii) minimise the risk of environmentally-driven deterioration;
(iii) have a minimal impact on the value(s) associated with the heritage;
(iv) be practically feasible to implement and maintain^9^.

However, uncertainties over future changes in climate^10–12^ pose significant challenges for selecting appropriate conservation strategies, because they may alter the processes causing environmentally-driven deterioration. There is therefore a pressing need for a research-driven approach to identify effective conservation strategies under both current and potential future climates.

Earthen heritage sites are commonly found in dryland environments, owing to a combination of widespread availability of earthen materials and favourable climatic conditions for their longevity^1^. In regions along the ancient Silk Road in Asia, rammed earth has been used to form a wide variety of structures, from homes and temples to entire cities^13–16^. These earthen sites have considerable historic and scientific value, because of the key role they play in documenting the spread of knowledge, religion and trade between Europe and Asia^9,17^.

The low durability of earthen sites when exposed to wet, windy conditions^16,18^ means that many earthen sites exhibit extensive deterioration features when they are not effectively conserved^4,8,15,19^. Two principal conservation approaches exist to minimise the rate at which the historical material deteriorates: either erodibility-reduction methods, which strengthen material, or erosivity-reduction methods, which reduce the strength of environmental drivers before they interact with a site. In general, many heritage experts express a strong preference for the latter – strategies that modify environmental conditions^9^. Potential conservation strategies for earthen heritage sites include nature-based solutions, such as windbreaks and greater natural vegetation coverage^9,20^.

In this paper, we present a framework to test the effectiveness of potential conservation strategies for earthen heritage under current and potential future climate scenarios. This framework is designed to empower decision makers to deploy appropriate conservation strategies quickly and efficiently in some of the most fragile sites on the planet.

## Modelling deterioration risk

This paper applies a novel numerical modelling method to evaluate the potential risk of deterioration under future climate and conservation scenarios. We use the Vegetation and Sediment TrAnsport model for Heritage Deterioration (ViSTA-HD), a cellular automaton model developed by the authors that couples environmental and heritage deterioration processes^21^.

ViSTA-HD simulates environmental conditions around earthen heritage sites in dryland environments and resolves deterioration risk in a spatial manner. This enables us to detect changes across wall faces under different climate and conservation scenarios. Richards et al.^21^ provide a detailed explanation and extensive set of verification and validation tests for ViSTA-HD, with empirical data supporting the model formulation. The “Methods” section in this paper provides further description of the model.

**Figure 1:**
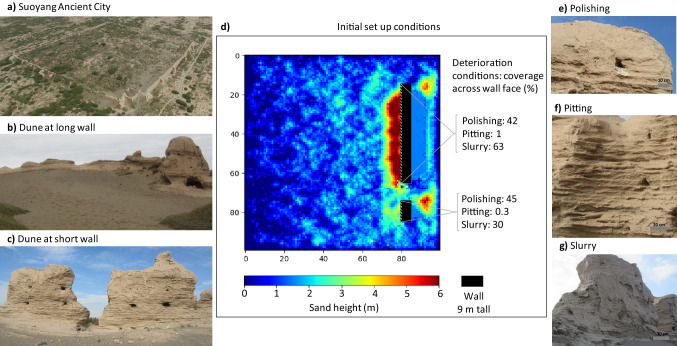
The site of Suoyang Ancient city (**a–c**), a plan view of the initial model setup (**d**) and the three major deterioration features (**e–g**).

In this study, we use ViSTA-HD to simulate the risk of deterioration over a 100-year time period at Suoyang Ancient City (锁阳城, hereafter Suoyang), an earthen heritage site located in semi-arid northwest China. Suoyang is an ancient rammed earth city built during the Han (206 BCE–220 CE) and Tang (618–907 CE) dynasties, and was enlisted as part of the Silk Roads World Heritage site in 2014^22^ (Fig. 1a). Many parts of the site, especially those facing the prevailing easterly wind, have experienced extensive deterioration^23^. On some walls perpendicular to the prevailing wind, dune features have also formed on wall faces both up and down wind (Fig. 1b,c). Twenty-first century climate projections for northwest China suggest sites, such as Suoyang, are facing future climate uncertainties. CMIP5^11,24^ and regional climate models (PRECIS^25,26^, RegCM3^27,28^, RegCM4^12^ and CMM5^27^), show rainfall projections ranging from a 10% decrease^10,12^ to 50% increase^11,12,28^, while wind velocity projections vary from a decrease of 1 ms^−1^ to an increase of 1 ms^−1^^27,29^.

To reflect conditions at Suoyang’s Inner City Wall, ViSTA-HD’s model space was populated with two walls: one 50 m in length and the other 10 m in length, and both 9 m high, reflecting the range of wall dimensions observed at the site. The walls were positioned perpendicular to the easterly prevailing wind (Fig. 1d). The modelled climatic and ecological conditions were reflective of present-day conditions at Suoyang unless otherwise stated and were informed by previous studies undertaken at the site^16,21^. Results of deterioration risk were similar for the two modelled walls, so we only present results from the 50 m wall in the main manuscript; results for the 10 m are provided in the Supplementary Information.

By resolving micro-scale environmental conditions occurring at the wall faces, including wind velocity and sediment movement, we used ViSTA-HD to model the risk of three dominant deterioration features at earthen heritage sites^16,23^: polishing, pitting and slurry. Polishing is caused by clean wind or sediment-laden wind smoothing the surface of the material^16^ (Fig. 1e). Pitting is caused by high-velocity or sediment-laden wind abrading the material and forming hollows^16,30^ (Fig. 1f). Slurry is caused by rain, and wind-driven rain, weakening the cementing material between particles, which results in material being transported down the wall face under gravity and forming a drape-like surface crust^8,16^ (Fig. 1g). The overall deterioration risk was calculated by averaging the risks of polishing, pitting and slurry. The values of modelled risk vary between 0 (representing very low risk) and 1 (very high risk). Analysable outputs from the model include wind velocity, sediment transport and rainfall across the model domain and the risks of deterioration across the wall face.

Given large uncertainties in the climate projections for northwest China^10^, we used a scenario-neutral approach to test the sensitivity of deterioration risk to possible future climates. We ran ViSTA-HD with three changes in mean annual rainfall (decrease of 10% and increases of 20% and 50%) and three in wind velocity (decrease of 1 ms^−1^ and increases of 2 ms^−1^ and 5 ms^−1^), relative to the current climatic conditions (Table 1). Two extreme scenarios were simulated to provide a range of possible heritage responses: a ‘low change’ scenario assumed a 10% decrease in rainfall and 1 ms^−1^ decrease in wind velocity, while a ‘high change’ scenario assumed a 50% increase in rainfall and 5 ms^−1^ increase in wind velocity (see Table 1 for details). Wind velocity and rainfall parameters were deliberately pushed to extremes to understand the system response under a wide variety of conditions.

**Table 1:** Climate and conservation scenarios modelled using ViSTA-HD. A decrease in conditions in shown with a ‘−’, an increase is shown with a ‘+’.

	Changes in mean annual wind velocity (ms^−1^) and rainfall (%)	Modelled climate scenarios: current climatic conditions at Suoyang							
		Extremes		Wind			Rain		
		− 1 (wind); − 10 (rain)	+ 5 (wind); + 50 (rain)	− 1	+ 2	+ 5	− 10	+ 20	+ 50
**Conservation scenarios**									
No conservation strategy	Control✓	✓	✓	✓	✓	✓	✓	✓	✓
80% vegetation cover randomly distributed	✓	✓	✓						
Windbreak 10 m tall, 60 m upwind	✓	✓	✓						
Windbreak 5 m tall, 30 m upwind	✓								
Two windbreaks (see above for dimensions)	✓								

We also tested the effectiveness of four nature-based conservation strategies in ViSTA-HD. Three strategies are based on different windbreak configurations (all formed of vegetation with 30% optical porosity), and the fourth involves increasing natural vegetation coverage to 80% (based on the most densely vegetated areas of Suoyang today) (Table 1). The two conservation strategies that caused the greatest reduction in deterioration risk under current climatic conditions were also run in combination with the low and high extreme climatic situations to test the effectiveness of the conservation strategies under a range of future climate scenarios (see Table 1).

For each climatic and conservation scenario, we performed a model spinup of 400 iterations, representing 100 years in real time. This ensured our modelled system was in dynamic equilibrium representative of conditions currently observed at Suoyang. The resultant environmental and deterioration conditions at the end of the spinup period were used as initial conditions for our model runs (Fig. 1d). The model was run for a further 400 iterations and the resultant deterioration risks were analysed and presented as our final results.

## Deterioration under future climate scenarios

**Figure 2:**
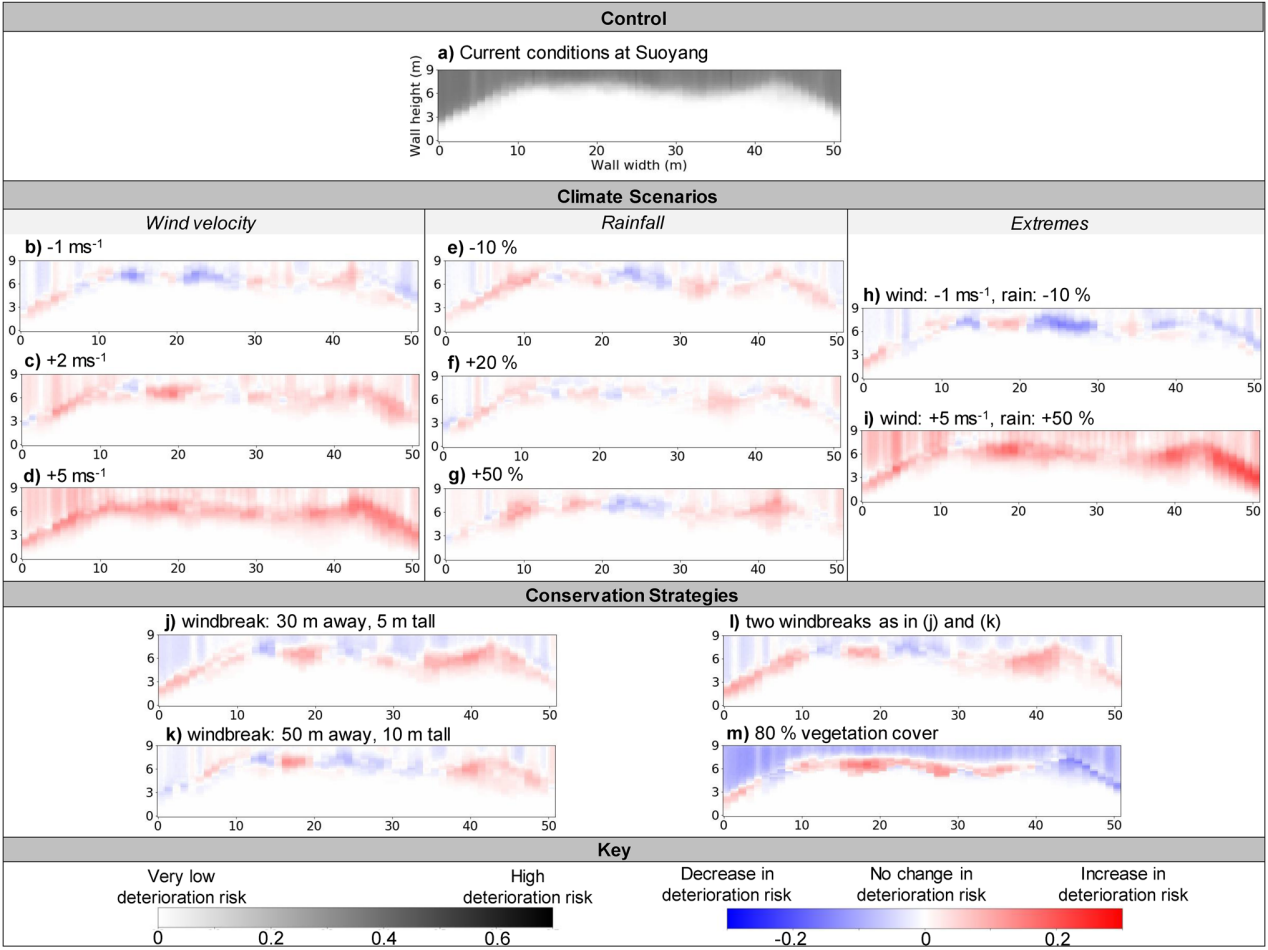
The overall risk of deterioration after 100 years under: (**a**) current climatic conditions; (**b**–**i**) different climatic changes; and (**j**–**m**) different conservation strategies. In panels (**b**–**m**), deviations from the control results are shown with red and blue, indicating an increased and decreased risk of deterioration, respectively.

Figure 2 shows the overall deterioration risk to earthen heritage after 100 years of exposure to future environmental conditions. Current conditions experienced at Suoyang were used to generate a control deterioration risk (Fig. 2a). This control was compared to the deterioration risk caused by potential future climatic (Fig. 2b–i) and conservation (Fig. 2j–m) scenarios.

Results indicate that exposed wall faces have a medium to high risk of deterioration, while areas covered with dunes have a notably lower risk (Fig. 2a). This implies that under present day conditions, earthen heritage sites are highly likely to continue experiencing environmentally-driven deterioration^16^, highlighting the urgent need for effective conservation strategies.

The lower risk of deterioration on areas below dune formations suggests that the development of dunes could act as a natural re-burial conservation strategy, despite sediment transport being associated with deterioration such as pitting^31,32^. Relying on re-burial as a conservation strategy may limit access to the site. However, partial re-burial can enable the plan form of the site to be legible, while minimising the formation of features such as hollowing at the base of walls^32^ . This strategy could also enhance the presentation of the ruined nature of the site^33^.

Under reduced wind velocities of 1 ms^−1^, the overall risk of deterioration generally decreased (Fig. 2b). Lower wind velocities resulted in lower rates of sediment entrainment^34^, minimising the risk of pitting across the wall face^19^ (Supplementary Fig. 6). However, the lower transportation rates also resulted in slower dune build-up over the wall face^34,35^. Consequently, a greater area of the wall face was exposed to environmental drivers of deterioration for a longer period of time, increasing the risk of both polishing and slurry in these areas (Supplementary Fig. 6). Above this area, a reduction in driving rain caused a decrease in the risk of slurry^36^ (Supplementary Fig. 6). As slurry tends to form surface crusts^5^, a decreased risk of slurry results in greater availability of material for polishing, which explains why the risk of polishing increased on the upper wall face despite lower mean annual wind velocities.

Under increased wind velocities (increases of 2 and 5 ms^−1^), the overall risk of deterioration increased substantially across the wall face (Fig. 2c,d). The increased wind velocities, increased rates of sediment transport^34^ and wind-driven rain^36^, resulted in higher risks of polishing, pitting and slurry across the wall face, especially with increases of 5 ms^−1^ in wind velocity (Supplementary Fig. 6).

Under the modelled changes in mean annual rainfall, the overall risk of deterioration remained relatively stable (Fig. 2e–g), although the relative dominance of deterioration features changed. Increased rainfall caused an increased risk of slurry, but formation of the crust associated with slurry reduced the risk of polishing^5^ (Supplementary Fig. 7). While changes in rainfall may only have a limited impact on the overall deterioration risk, changes in the relative dominance of individual features could alter the visual aesthetics of earthen heritage sites. Therefore, if future rainfall changes, a values-based judgement would be required^9^ to assess if the aesthetic impacts to the site were deemed acceptable.

Finally, combined climatic changes in mean wind velocity and annual rainfall had a notably greater impact on deterioration risk than when climatic changes occurred individually (Fig. 2h–i). Consequently, conservation strategies need to remain effective even if multiple climatic changes occur concurrently.

## Effectiveness of nature-based conservation strategies

To assess the extent to which nature-based conservation strategies minimise deterioration risk under current climate conditions, we tested four conservation strategies using ViSTA-HD: (1) a 5 m-high windbreak located 30 m away from the wall; (2) a 10 m-high windbreak located 60 m away from the wall; (3) two windbreaks together, as described in (1) and (2); and (4) 80% randomly distributed vegetation cover (up to 1.5 m tall). The deterioration risk for each conservation scenario (Fig. 2k–m) was compared to the control risk of deterioration where no conservation strategy was implemented (Fig. 2a).

The three windbreak configurations had a similar effect on the overall risk of deterioration (Fig. 2k–l). Since windbreaks reduce wind velocities and trap sediment in their lee^37,38^, dunes built up more slowly against our simulated wall face. Consequently, the deterioration risk increased above the dune formation zone due to the slower build-up of sediment^39^, but decreased at the wall top due to lower wind velocities^19^. Vegetation cover of 80% caused a greater reduction in the overall risk of deterioration than the windbreaks (Fig. 2m). This suggests that under current conditions, increasing the natural vegetation cover is likely to be more effective at reducing deterioration risk at earthen sites like Suoyang than planting windbreaks. However, if a windbreak was preferable for certain reasons (e.g. wanting a strategy implemented within a contained space), shorter windbreaks would likely to be less visually intrusive on the aesthetics of the site and could be constructed using native shrubs rather than non-native trees, which are often unsuitable for the soil conditions and climate in semi-arid areas^40^.

**Figure 3:**
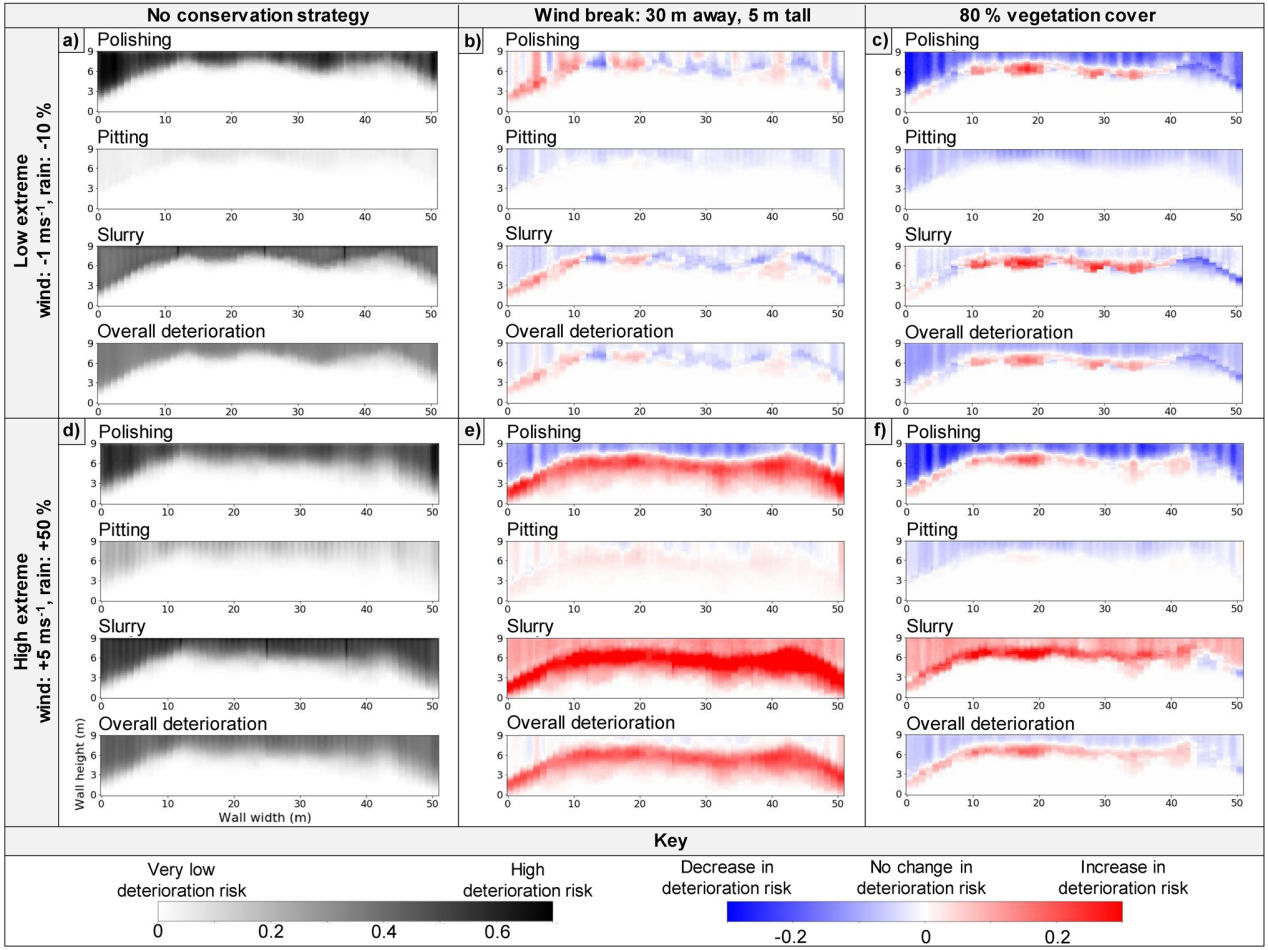
The deterioration risk and difference in deterioration risk for polishing, pitting, slurry and the overall deterioration after 100 years of model time, under (**a**–**c**) a regime of low mean annual wind velocity and rainfall, and (**d**–**f**) a regime of high wind velocity and rainfall. Different conservation strategies were applied: (**a**,**d**) no conservation strategy (control); (**b**,**e**) addition of a windbreak; and (**c**,**f**) 80% vegetation cover.

Given that nature-based conservation strategies tend to be implemented with the intent of lasting multiple decades, these strategies need to remain effective under potential future climatic scenarios. Because of their effectiveness at minimising deterioration under current conditions, the short windbreak and 80% vegetation coverage interventions were tested under the low and high extreme future climatic scenarios. The deterioration risk was compared to the risk of deterioration under the low/high extreme future climatic scenarios, respectively, where no conservation strategy was implemented (Fig. 3).

When subjected to low and high extreme future climatic scenarios, 80% vegetation coverage was more effective than the windbreak at minimising polishing, pitting and the overall deterioration (Fig. 3). The risk of slurry increased with the implementation of either conservation strategy. This increase occurred due to lower wind velocities reducing the risk of polishing and pitting, which in turn meant more earthen material was exposed to rain-driven deterioration^16^ (Fig. 3e,f). The increased risk of slurry under the 80% vegetation coverage intervention was counterbalanced by the decreases in polishing and pitting risks, resulting in a general decrease in overall deterioration across the wall face. This was the opposite to the windbreak, which caused an overall increase in deterioration risk.

## A research-driven future for earthen heritage

ViSTA-HD provides users with the ability to iteratively assess the benefits and trade-offs of conservation decisions in the face of climatic uncertainties. As climate conditions and site deterioration change, model inputs and projects can be easily altered to provide dynamic management advice. From our application of ViSTA-HD to conditions at Suoyang, we found that increasing vegetation coverage is likely to be the most effective strategy for decreasing deterioration risk under both current and future climatic scenarios. Our results suggest this strategy is relevant to a wide variety of possible climatic scenarios. We urge conservation scientists to pursue similar strategies of combining field data with modelling to assess deterioration risk and the potential effects of their planned interventions. This approach has great potential for decreasing deterioration risk at earthen heritage sites in dryland regions around the world.

## Methods: ViSTA-HD model

The Vegetation and Sediment TrAnsport model for Heritage Deterioration (ViSTA-HD) is a coupled cellular automaton (CA) model designed to simulate the risk of environmentally driven deterioration on earthen heritage. ViSTA-HD is based on the original ViSTA model, which was designed to simulate the evolution of semi-vegetated dryland landscapes^41,42^.

**Figure 4:**
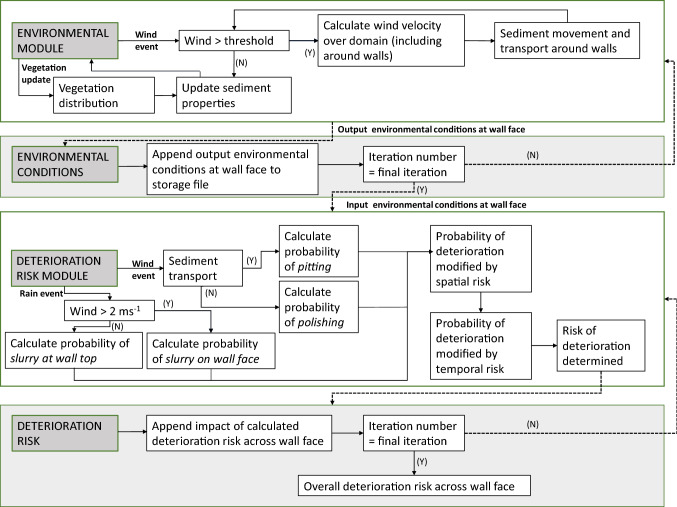
A schematic of the environmental and deterioration risk modules within ViSTA-HD. Reproduced with permission^21^.

ViSTA-HD is comprised of two modules (an environmental module and a deterioration module) that interact at each timestep in the model (Fig. 4). The environmental module simulates sediment transport dynamics around earthen walls by spatially resolving wind velocity, rainfall and sediment transport across a horizontal plane. Environmental conditions occurring at the upwind face of the wall are stored and used as the input conditions in the deterioration module. The deterioration module spatially determines the risk of three deterioration features, polishing, pitting and slurry, commonly found on earthen heritage across the wall face as well as the overall deterioration risk.

Both the environmental and deterioration modules divide the model domain into discrete uniform grid cells. Each grid cell holds a number of properties (e.g. vegetation characteristics and height above the deposited sand, for the environmental and deterioration module, respectively). Basic rules are applied to each cell and, depending on the cell’s properties, local neighbourhood operations allow dynamic responses to emerge. Empirical field data collected in semi-arid environments^16,43–45^ was used to parameterise the basic rules where possible. The properties of each cell update at the end of every timestep. The state of a cell at the end of a timestep is used as the initial state of the cell at the next timestep.

### Environmental module

The environmental module controls the wind dynamics and sediment movement. Horizontal wind velocity is inputted at the edge of the model grid space. It interacts with vegetation and wall elements within the model, with the initial decrease in velocity and downwind distance for wind recovery being dependent of the element’s height and porosity^41,43,44^. In addition, up-wind of the walls, wind velocity is reduced due to the occurrence of turbulent eddies. Airflow acceleration/deceleration is simulated over surface topography such as dune formation.

The sediment flux is a function of the wind velocity. The volume of transported sediment is determined by probabilities of erosion and deposition which are dependent on surface characteristics including the presence of vegetative and wall elements in the model space.

Further details on the environmental module is provided by Mayaud et al.^41^.

### Deterioration module

The deterioration module determines the risk of deterioration across the wall face for polishing, pitting, slurry and overall deterioration. The risk of deterioration is considered to be the likelihood of deterioration occurring in a given space—not the magnitude of deterioration caused. The modelled risk values vary between very low risk (0) and very high risk (1). The risk of deterioration is determined using three factors: (1) the environmental conditions impacting on the given area of the wall; (2) the extent to which areas around the given area of the wall also experience the deterioration; and (3) previous deterioration on the given area of the wall.

More details on the deterioration module can be found in Richards et al.^21^.

### Model runs

ViSTA-HD was used to assess the risk of deterioration: (1) under current conditions experienced at Suoyang, (2) under potential future climate scenarios and, (3) with the implementation of selected conservation strategies (Table 1). For all simulations, the model was run for 400 iterations, representing 100 years (one iterations = 3 months). Due to the inherent variability in ViSTA-HD, each module was run until a stabilisation of the means had occurred. Consequently, for each scenario, the environmental module was run 10 times. Then for each run of the environmental module, the deterioration module was run 20 times (resulting in 200 runs of the deterioration module for each scenario). The mean and standard deviation of deterioration risk was calculated for each cell in the model domain for each deterioration feature.

All model runs were implemented using code written in the Python programming language by the authors. The model code is openly available from the Oxford University Research Archive (https://doi.org/10.5287/bodleian:nr86P0OjY).

### Input conditions

Wind velocity and rainfall input conditions were based on meteorological data collected by an automatic weather station at Suoyang and historic records provided by the Dunhuang Academy (unpublished). At each iteration, wind velocities and rainfall volume were randomly selected from probability density functions representative of the seasonal conditions at Suoyang. Changes to the mean annual wind velocity and mean annual rainfall were implemented by decreasing/increasing the wind velocity probability density function.

Windbreaks were implemented within ViSTA-HD by placing vegetation elements with 30% porosity in a 3 m wide strip upwind of the walls. This porosity was chosen as windbreak studies have shown this to create the largest downwind zone of reduced wind velocities^37,46^. Eighty percent vegetation coverage was implemented by increasing the proportion of randomly distributed vegetation elements in the model space. In all model runs, vegetation was assumed to remain the stable as at Suoyang image analysis has shown vegetation cover to remain stable over a decadal period^21^.

### Model implementation

We propose that findings from ViSTA-HD simulations should be implemented at earthen heritage sites using an iterative framework, which integrates field data collection with modelling approaches. Data collection is required to ensure ViSTA-HD is initialised with site-specific climatic and environmental conditions. This ensures that the modelled deterioration risks are site-specific and relevant to decision-makers.

The suite of deterioration risks produced by ViSTA-HD can then be used to assess the relative benefits and trade-offs between conservation strategies in the context of: (1) a strategy’s ability to minimise deterioration risk, as well as, (2) other strategic factors, such as the implementation and maintenance costs, and the size and the aesthetic impact of a strategy^9^. Field tests using the chosen conservation strategy should be carried out before implementation. This would ideally be undertaken over a minimum of 1 year to ensure the desired impact on deterioration can be achieved, without unforeseen consequences, before applying similar approaches to entire sites.

Once field tests produce the desired results, the implementation of the conservation strategy can be initiated. As this framework is iterative, ongoing monitoring of environmental processes at, and deterioration condition of, the site enables the impact of these strategies to be monitored and adjusted if required.

### Model considerations

As with all models, ViSTA-HD cannot capture all parts of the earth system. It focuses on the risk of deterioration driven by environmental processes given the preference expressed by earthen heritage experts for conservation strategies to modify the environment rather than the historic material. Consequently, ViSTA-HD does not include other factors, such as material characteristics, which will also affect the absolute volume of deteriorated material. Therefore, in no circumstances should ViSTA-HD be used to ‘guarantee’ that an area will be at no risk of deterioration, instead it should be used as a research tool to aid understandings of how the environment poses risks to earthen heritage sites.

## Supplementary information

Supplementary Figures.

## References

[1] WHEAP. *World Heritage: Inventory of Earthen Architecture*. (2012).

[2] Zhang Y, Ye WM, Chen B, Chen YG, Ye B (2016). Desiccation of NaCl-contaminated soil of earthen heritages in the Site of Yar City, northwest China. Appl. Clay Sci..

[3] Cui K, Wu G, Du Y, An X, Wang Z (2019). The coupling effects of freeze-thaw cycles and salinization due to snowfall on the rammed earth used in historical freeze-thaw cycles relics in northwest China. Cold Reg. Sci. Technol..

[4] Du Y (2017). A model characterizing deterioration at earthen sites of the Ming Great Wall in Qinghai Province, China. Soil Mech. Found. Eng..

[5] Cui K, Du Y, Zhang Y, Wu G, Yu L (2019). An evaluation system for the development of scaling off at earthen sites in arid areas in NW China. Herit. Sci..

[6] Matero F, Moss E (2004). Temporary site protection for earthen walls and murals at Çatalhöyük, Turkey. Conserv. Manag. Archaeol. Sites.

[7] Oliver, A., Getty Adobe Project, Getty Conservation Institute & Museum of New Mexico. In *Fort Selden Adobe Test Wall Project: Phase I: Final Report* (2000).

[8] Shao M, Li L, Wang S, Wang E, Li Z (2013). Deterioration mechanisms of building materials of Jiaohe ruins in China. J. Cult. Herit..

[9] Richards J (2018). Finding common ground between United Kingdom based and Chinese approaches to earthen heritage conservation. Sustainability.

[10] Christensen JH, Stocker TF (2013). Climate phenomena and their relevance for future regional climate change. Climate Change 2013: The Physical Science Basis. Contribution of Working Group I to the Fifth Assessment Report of the Intergovernmental Panel on Climate Change.

[11] Wang L, Chen W (2014). A CMIP5 multimodel projection of future temperature, precipitation, and climatological drought in China. Int. J. Climatol..

[12] Hui P (2018). Climate change projections over China using regional climate models forced by two CMIP5 global models. Part II: projections of future climate. Int. J. Climatol..

[13] Li Z (2011). Conservation of Jiaohe ancient earthen site in China. J. Rock Mech. Geotech. Eng..

[14] Li L, Shao M, Wang S, Li Z (2011). Preservation of earthen heritage sites on the Silk Road, northwest China from the impact of the environment. Environ. Earth Sci..

[15] Pu T, Chen W, Du Y, Li W, Su N (2016). Snowfall-related deterioration behavior of the Ming Great Wall in the eastern Qinghai–Tibet Plateau. Nat. Hazards.

[16] Richards J, Zhao G, Zhang H, Viles H (2019). A controlled field experiment to investigate the deterioration of earthen heritage by wind and rain. Herit. Sci..

[17] Xie Y, Ward R, Fang C, Qiao B (2007). The urban system in West China: a case study along the mid-section of the ancient Silk Road–He-Xi Corridor. Cities.

[18] Beckett CTS, Jaquin PA, Morel J-C (2020). Weathering the storm: a framework to assess the resistance of earthen structures to water damage. Constr. Build. Mater..

[19] Wang XD, Zhang HY, Yan GS, Pei QQ (2010). Durability of ancient earthen architecture under wind erosion in the Milan Ancient City along the Silk Road of China. Adv. Mater. Res..

[20] Li GS, Qu JJ, Han QJ, Fang HY, Wang WF (2013). Responses of three typical plants to wind erosion in the shrub belts atop Mogao Grottoes, China. Ecol. Eng..

[21] Richards, J. *et al.* Modelling the risk of deterioration at earthen heritage sites in drylands. *Earth Surf. Process. Landforms* Ahead of print (2020).

[22] UNESCO. *Silk Roads: Initial Section of the Silk Roads, the Routes Network of Tian-shan Corridor. Nomination Document* (2014).

[23] Richards J, Viles H, Guo Q (2020). The importance of wind as a driver of earthen heritage deterioration in dryland environments. Geomorphology.

[24] Zhou B, Wen QH, Xu Y, Song L, Zhang X (2014). Projected changes in temperature and precipitation extremes in China by the CMIP5 multimodel ensembles. J. Clim..

[25] Guo J, Huang G, Wang X, Li Y, Lin Q (2017). Investigating future precipitation changes over China through a high-resolution regional climate model ensemble. Earth’s Futur..

[26] Guo J, Huang G, Wang X, Li Y, Yang L (2018). Future changes in precipitation extremes over China projected by a regional climate model ensemble. Atmos. Environ..

[27] Jiang Y (2010). Projections of wind changes for 21st century in China by three regional climate models. Chin. Geogr. Sci..

[28] Gao X, Shi Y, Zhang D, Giorgi F (2012). Climate change in China in the 21st century as simulated by a high resolution regional climate model. Chin. Sci. Bull..

[29] Xiong Y, Xin X, Kou X (2019). Simulation and projection of near-surface wind speeds in China by BCC-CSM Models. J. Meteorol. Res..

[30] Mao X, Zhoa D, Zhang W (2015). Experimental study on the effects of wind erosion and reinforcement for the rammed soil. J. Xi’an Univ. Archit. Technol. Nat. Sci. Ed..

[31] Agnew N, Selwitz C, Demas M (2004). Reburial research: preliminary field experiments at Fort Selden. Conserv. Manag. Archaeol. Sites.

[32] Woolfitt C, Ashurst J (2007). Preventive conservation of ruins: reconstruction, reburial and enclosure. Conservation of Ruins.

[33] Douglas-Jones R, Hughes JJ, Jones S, Yarrow T (2016). Science, value and material decay in the conservation of historic environments. J. Cult. Herit..

[34] Dong Z, Liu X, Wang H, Wang X (2003). Aeolian sand transport: a wind tunnel model. Sediment. Geol..

[35] Tsoar H (1983). Wind tunnel modeling of echo and climbing dunes. Dev. Sedimentol..

[36] Blocken B, Carmeliet J (2004). A review of wind-driven rain research in building science. J. Wind Eng. Ind. Aerodyn..

[37] Santiago JL, Martín F, Cuerva A, Bezdenejnykh N, Sanz-Andrés A (2007). Experimental and numerical study of wind flow behind windbreaks. Atmos. Environ..

[38] Cornelis WM, Gabriels D (2005). Optimal windbreak design for wind-erosion control. J. Arid Environ..

[39] Oliver A, Avrami EC, Guillaud H, Hardy M (2008). Conservation of earthen archaeological sites. Terra Literature Review: An Overview of Research in Earthen Architecture Conservation.

[40] Cao S (2011). Greening China naturally. Ambio.

[41] Mayaud JR, Bailey RM, Wiggs GFS (2017). A coupled vegetation/sediment transport model for dryland environments. J. Geophys. Res. Earth Surf..

[42] Mayaud JR, Bailey RM, Wiggs GFS (2017). Modelled responses of the Kalahari Desert to 21st century climate and land use change. Nat. Sci. Reports.

[43] Mayaud JR, Wiggs GFS, Bailey RM (2016). Dynamics of skimming flow in the wake of a vegetation patch. Aeolian Res..

[44] Mayaud JR, Wiggs GFS, Bailey RM (2016). Characterizing turbulent wind flow around dryland vegetation. Earth Surf. Process. Landforms.

[45] Mayaud JR, Wiggs GFS, Bailey RM (2017). A field-based parameterization of wind flow recovery in the lee of dryland plants. Earth Surf. Process. Landforms.

[46] Heisler GM, Dewalle DR (1988). Effects of windbreak structure on wind flow. Agric. Ecosyst. Environ..

