# Supplementary material for: Deterioration risk of dryland earthen heritage sites facing future climatic uncertainty
Source: Sci Rep. 2020 Oct 2;10:16419. doi: 10.1038/s41598-020-73456-8 (PMC7532530; doi:10.1038/s41598-020-73456-8)
Supplement: Supplementary file 1 — Supplementary Figures. [file 41598_2020_73456_MOESM1_ESM.docx]

**Supplementary Information for:**

**Deterioration risk of dryland earthen heritage sites facing future climatic uncertainty**

Jenny Richards^1*^, Richard Bailey^1^, Jerome Mayaud^1^, Heather Viles^1^, Qinglin Guo^2^, Xudong Wang^3^

^1^School of Geography and the Environment, University of Oxford, Oxford, OX1 3QF

^2^Dunhuang Academy, Dunhuang 736200, China

^3^The Palace Museum, Beijing, 100009, China

^*^Corresponding author: [Jennifer.richards@ouce.ox.ac.uk](mailto:Jennifer.richards@ouce.ox.ac.uk)


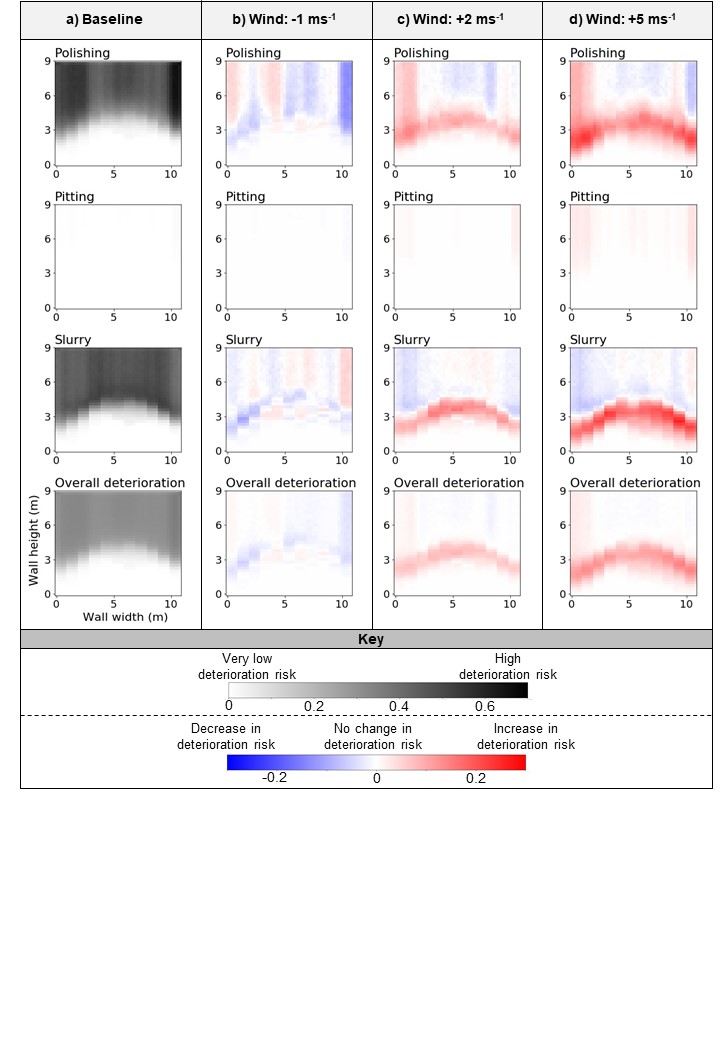


**Supplementary Figure 1:** The risk of polishing, pitting, slurry and overall deterioration after 100 years of model time on a 10 m long wall, **(a)** under climatic conditions representative of those currently at Suoyang and **(b-d)** the resultant change in deterioration risk from a: **(b)** 1 ms^-1^ decrease, **(c)** 2 ms^-1^ increase and **(d)** 5 ms^-1^ increase, in mean annual wind velocity.


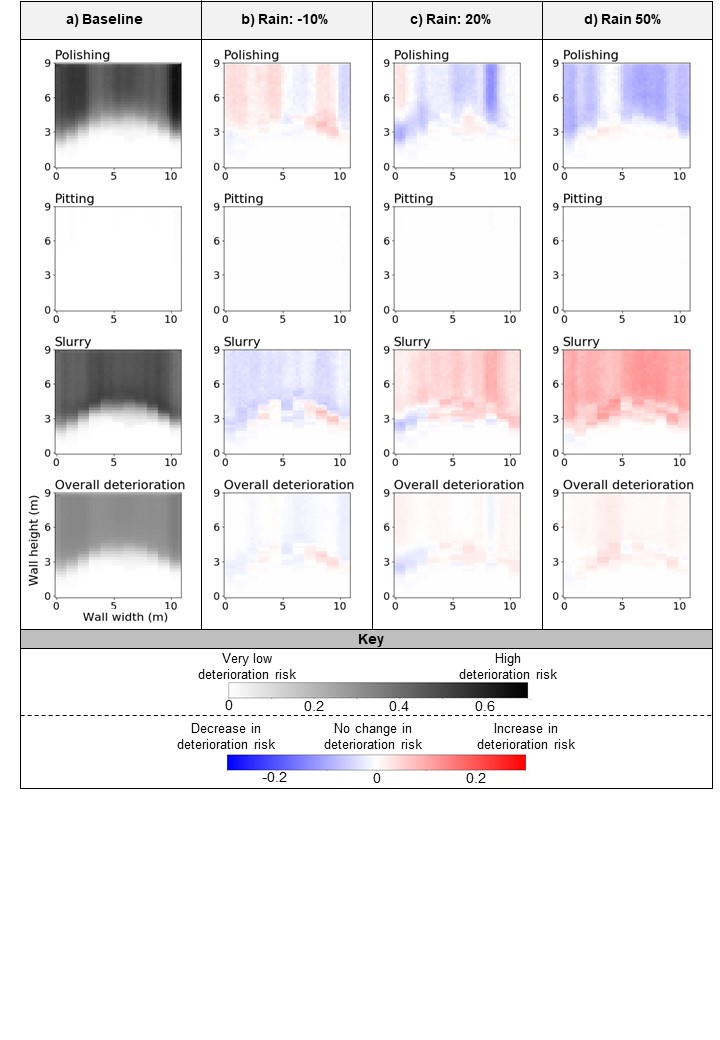


**Supplementary Figure 2:** The risk of polishing, pitting, slurry and overall deterioration after 100 years of model time on a 10 m long wall, **(a)** under climatic conditions representative of those currently at Suoyang and **(b-d)** the resultant change in deterioration risk from a: **(b)** 10% decrease, **(c)** 20% increase and **(d)** 50% increase in mean annual rainfall.


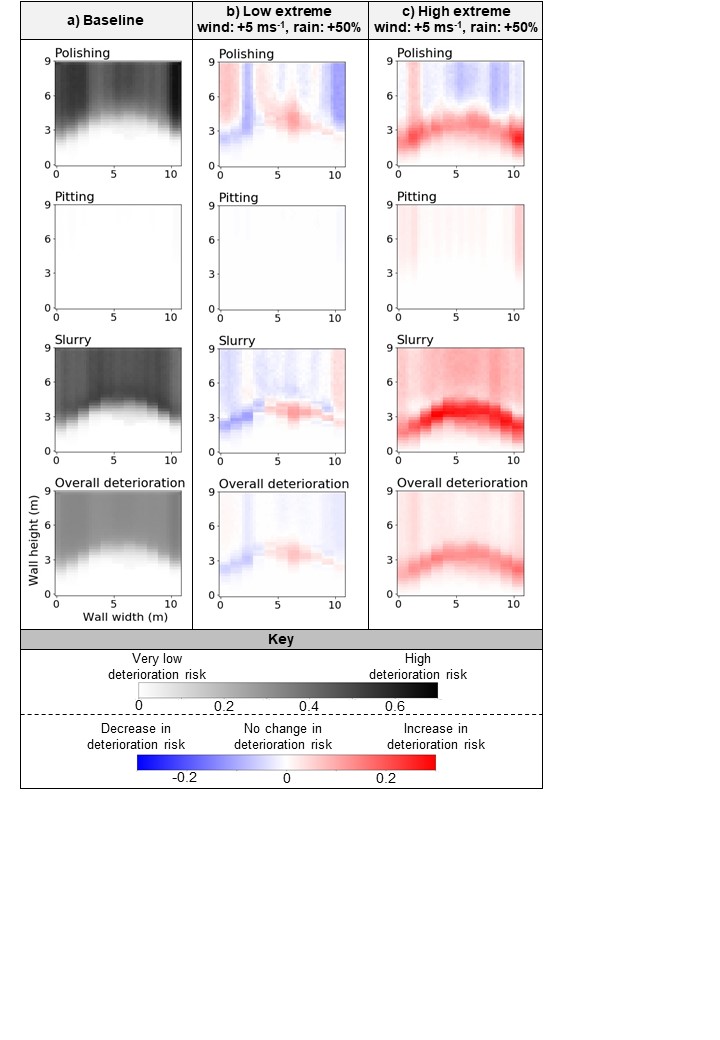


**Supplementary Figure 3:** The risk of polishing, pitting, slurry and overall deterioration after 100 years of model time on a 10 m long wall, **(a)** under climatic conditions representative of those currently at Suoyang and **(b-d)** the resultant change in deterioration risk from a: **(b)** 1 ms^-1^ decrease and 10% decrease, **(c)** 5 ms^-1^ increase and 50% increase, in mean annual wind velocity and mean annual rainfall, respectively.


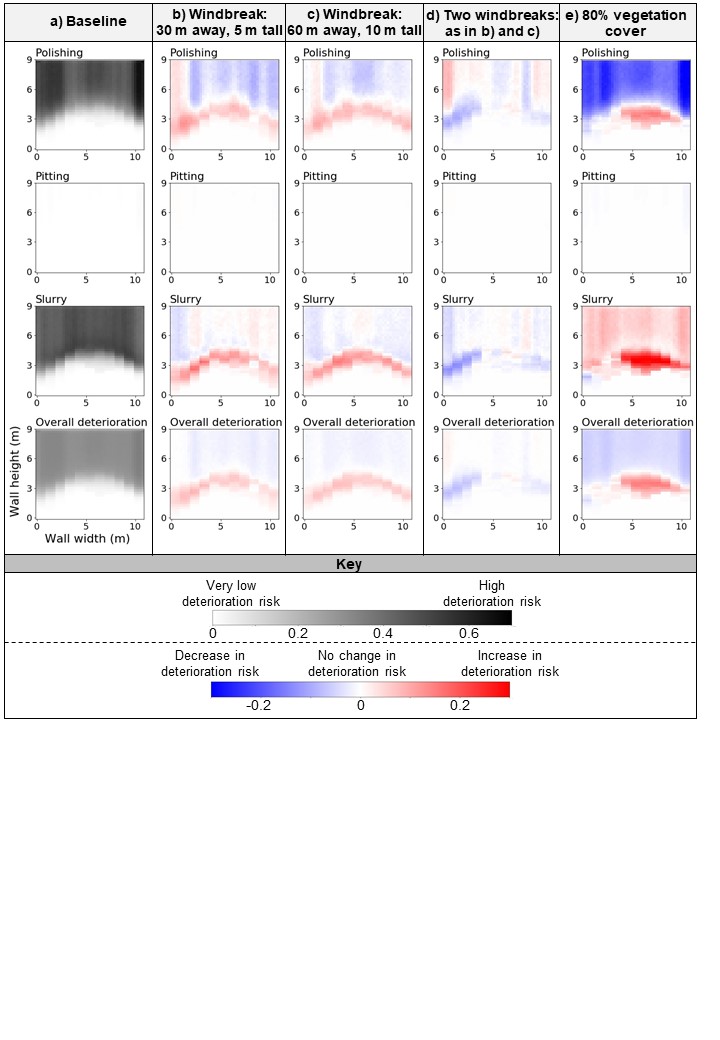


**Supplementary Figure 4:** The risk of polishing, pitting, slurry and overall deterioration after 100 years of model time on a 10 m long wall, **(a)** under climatic conditions representative of those currently at Suoyang and **(b-d)** the resultant change in deterioration risk from modelled conservation strategies **(b)** a near windbreak, **(c)** a far windbreak and **(d)** two windbreaks and **(e)** 80% vegetation coverage.


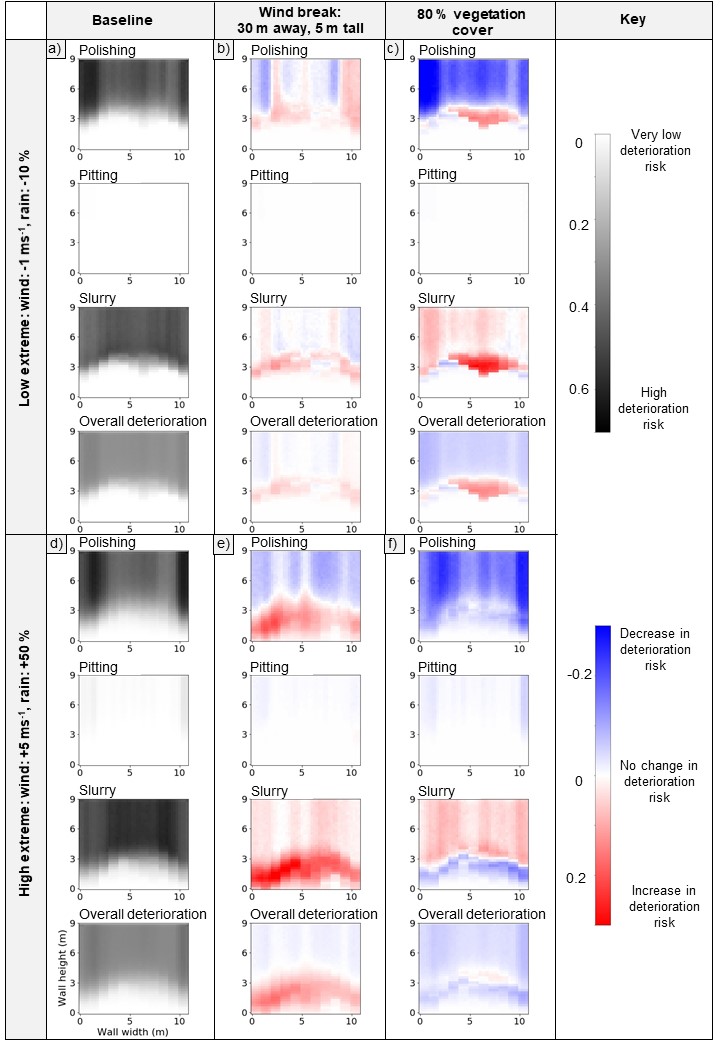


**Supplementary Figure 5:** The deterioration risk and difference in deterioration risk for polishing pitting, slurry and overall deterioration after 100 years of model time on a 10 m long wall, under **(a-c)** a regime of low mean annual windspeed and rainfall, and **(d-f)** a regime of high windspeed and rainfall. Different conservation strategies were applied: **(a, d)** no conservation strategy (baseline); **(b, e)** addition of a windbreak and **(c, f)** 80% vegetation cover.


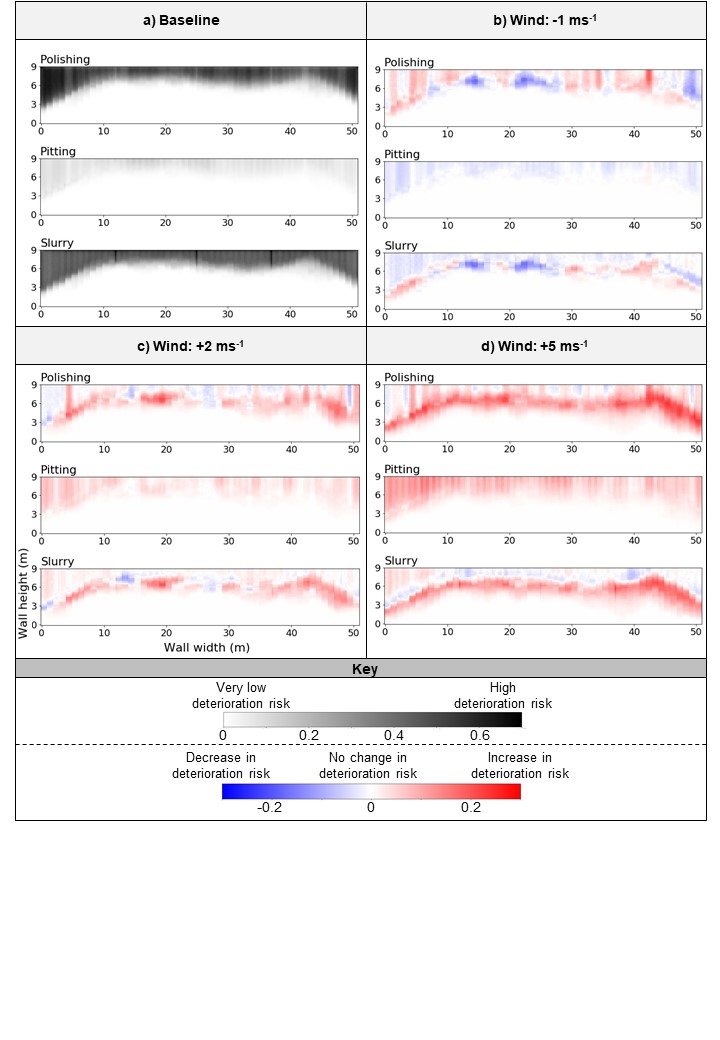


**Supplementary Figure 6:** The risk of polishing, pitting and slurry after 100 years of model time on a 50 m long wall, **(a)** under climatic conditions representative of those currently at Suoyang and **(b-d)** the resultant change in deterioration risk from a: **(b)** 1 ms^-1^ decrease, **(c)** 2 ms^-1^ increase and **(d)** 5 ms^-1^ increase, in mean annual wind velocity.


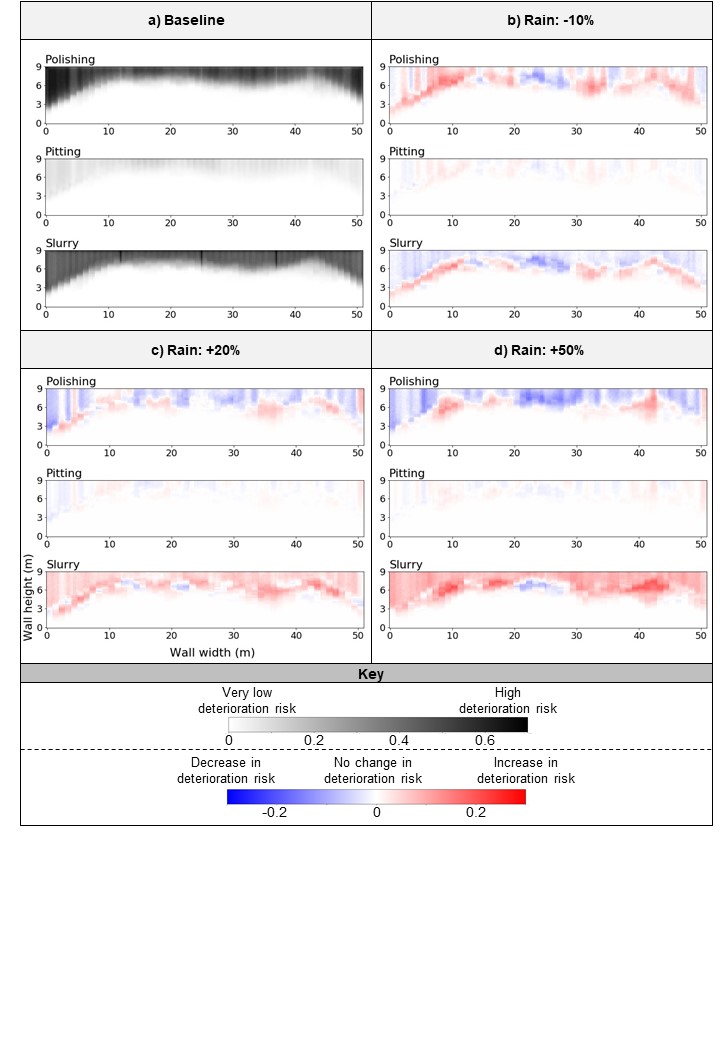


**Supplementary Figure 7:** The risk of polishing, pitting and slurry after 100 years of model time on a 50 m long wall, **(a)** under climatic conditions representative of those currently at Suoyang and **(b-d)** the resultant change in deterioration risk from a: **(b)** 10% decrease, **(c)** 20% increase and **(d)** 50% increase in mean annual rainfall.


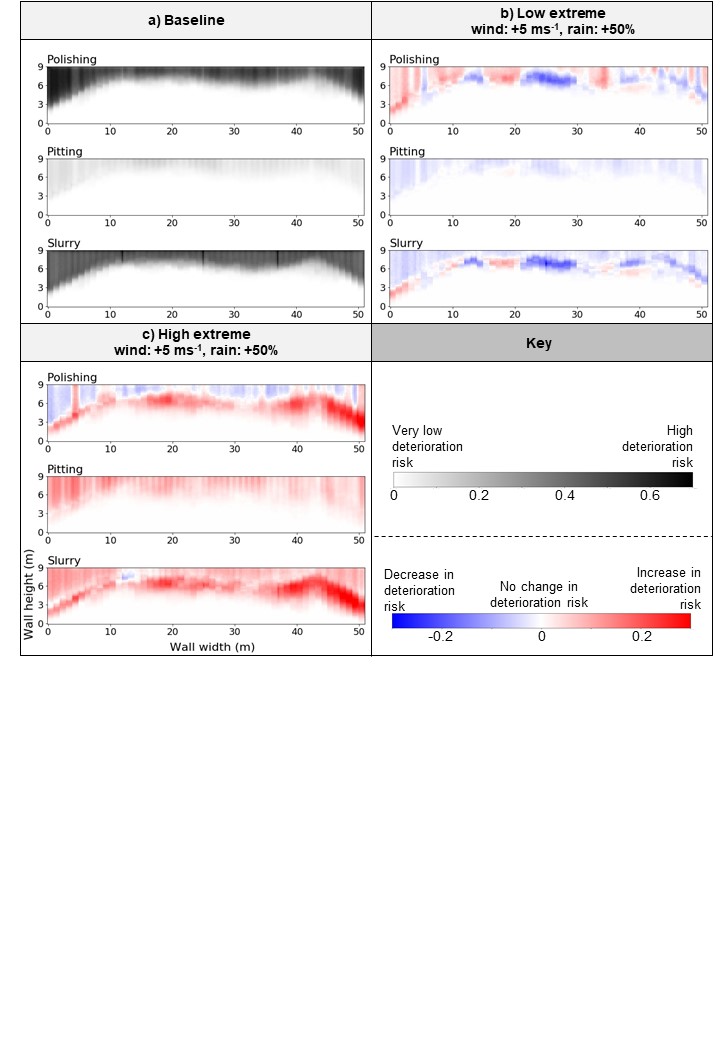


**Supplementary Figure 8:** The risk of polishing, pitting and slurry after 100 years of model time on a 50 m long wall, **(a)** under climatic conditions representative of those currently at Suoyang and **(b-d)** the resultant change in deterioration risk from a: **(b)** 1 ms^-1^ decrease and 10% decrease, **(c)** 5 ms^-1^ increase and 50% increase, in mean annual wind velocity and mean annual rainfall, respectively.


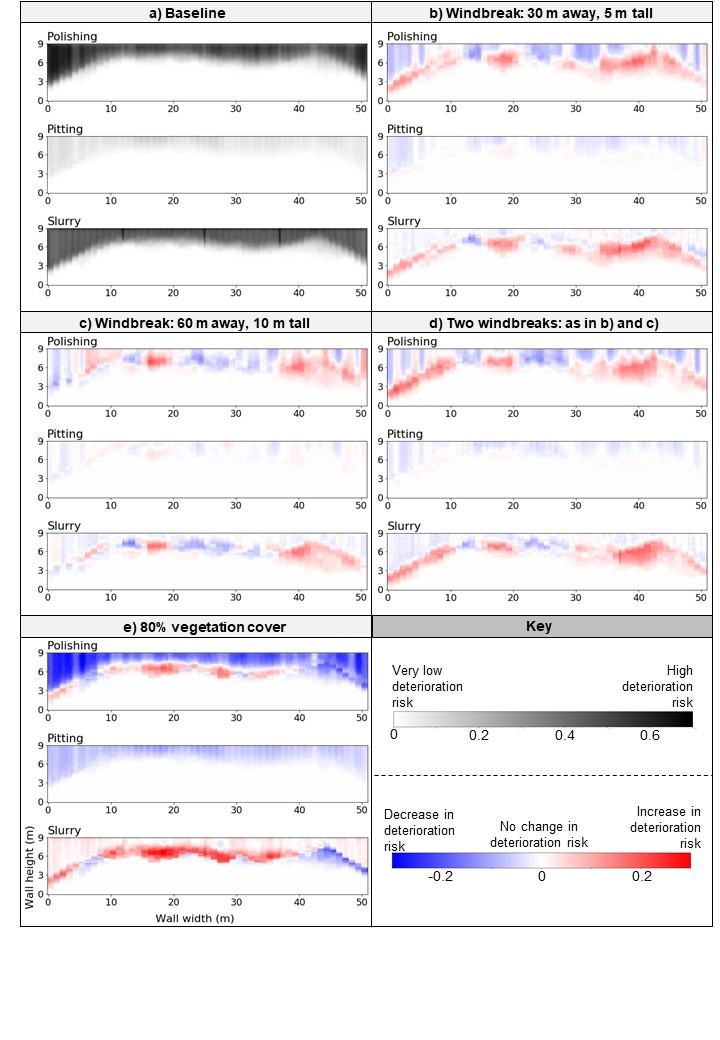


**Supplementary Figure 9:** The risk of polishing, pitting and slurry after 100 years of model time on a 50 m long wall, **(a)** under climatic conditions representative of those currently at Suoyang and **(b-d)** the resultant change in deterioration risk from modelled conservation strategies **(b)** a near windbreak, **(c)** a far windbreak and **(d)** two windbreaks and **(e)** 80% vegetation coverage.
